# The Feasibility of a Primary Care Based Navigation Service to Support Access to Health and Social Resources: The Access to Resources in the Community (ARC) Model

**DOI:** 10.5334/ijic.6500

**Published:** 2022-11-22

**Authors:** Simone Dahrouge, Alain P. Gauthier, Francois Durand, Manon Lemonde, Kiran Saluja, Claire Kendall, Kamila Premji, Justin Presseau, Marie-Hélène Chomienne, Darene Anne Toal-Sullivan, Patrick Timony, Andrea Perna, Denis Prud’homme

**Affiliations:** 1 Bruyère Research Institute, Ottawa, ON, Canada; 2 Department of Family Medicine, University of Ottawa, Ottawa, ON, Canada; 3 School of Kinesiology and Health Sciences, Laurentian University, Sudbury, ON, Canada; 4 Telfer School of Management, University of Ottawa, Ottawa, ON, Canada; 5 Institut du Savoir Montfort, Ottawa, ON, Canada; 6 Faculty of Health Sciences, Ontario Tech University, Oshawa, ON, Canada; 7 The Ottawa Hospital Research Institute, Ottawa, ON, Canada; 8 Department of Family Medicine, Western University, Ontario, Canada; 9 Centre for Rural and Northern Health Research, Laurentian University, Sudbury, ON, Canada; 10 Université de Moncton, Moncton, New Brunswick, Canada

**Keywords:** Feasibility, patient-navigation, integrated-care, social-prescribing, community resources

## Abstract

**Introduction:**

We established a patient centric navigation model embedded in primary care (PC) to support access to the broad range of health and social resources; the Access to Resources in the Community (ARC) model.

**Methods:**

We evaluated the feasibility of ARC using the rapid cycle evaluations of the intervention processes, patient and PC provider surveys, and navigator log data. PC providers enrolled were asked to refer patients in whom they identified a health and/or social need to the ARC navigator.

**Results:**

Participants: 26 family physicians in four practices, and 82 of the 131 patients they referred. ARC was easily integrated in PC practices and was especially valued in the non-interprofessional practices. Patient overall satisfaction was very high (89%). Sixty patients completed the post-intervention surveys, and 33 reported accessing one or more service(s).

**Conclusion:**

The ARC Model is an innovative approach to reach and support a broad range of patients access needed resources. The Model is feasible and acceptable to PC providers and patients, and has demonstrated potential for improving patients’ access to health and social resources. This study has informed a pragmatic randomized controlled trial to evaluate the ARC navigation to an existing web and telephone navigation service (Ontario 211).

## Background

Primary care is the coordination hub for medical services and plays a large role in helping patients adopt healthy behaviours to thwart disease and prevent the progression of existing ones [1,2,3,4,5,6,7]. Strategically, it is the ideal health care sector in which to embed intervention on the social determinants of health (SDoH) and redress inequities that stem from social disadvantage by supporting access to needed health and social services [8,9]. These include programs that address income security, affordable housing, and social isolation [10,11,12,13,14], and programs that aim to prevent the onset of disease or the progression of an existing condition through health behaviour modification including smoking cessation programs, and those promoting physical activity and healthy diets [15,16,17,18,19,20,21]. This is of great importance because healthy behaviours and SDoH are estimated to account for 70% of health outcomes [22]. The relationship primary care providers (PCPs) have with their patients and the trust they have established facilitates the identification of patient’s health and social needs [10,23,24], and is an important contributor to a patient’s motivation for engagement in self-care [25].

Many programs aiming to address health and social needs are available, but patients will often face barriers that will prevent them to access these resources, including those stemming from poor awareness of existing services, affordability, need for physical accommodations, and or lack of confidence [26,27,28]. However, most primary care practices do not have sufficient information or capacity to provide the assistance required to achieve access to overcome barriers and access the needed resources [29,30,31]. Navigation services have been shown to provide the support patients need to overcome access barriers, offers an approach for integrated care, thereby reducing health inequities [32,33,34]. However, most existing navigation models focus on medically complex patients [35,36,37,38,39], or targeted populations defined by their health conditions (e.g., cancer, diabetes) [40,41,42] or socio-cultural profile such as new immigrants or children [40,43,44,45]. These targeted approaches limit reach and fail to recognize the multi-dimensionality of the individual.

In 2018, we established and studied a whole-person, patient-centric navigation model embedded in primary care to achieve broad population reach and support practice patients access the needed health and social resources; the Access to Resources in the Community (ARC) [46]. This study was conducted under the IMPACT (Innovative Models Promoting Access-to-Care Transformation) international study, which addressed priority gaps in equitable access to primary health care [47]. In this paper we describe the feasibility of the ARC Model across seven focus areas: demand, implementation, adaptation, integration, practicality, acceptability, and potential for efficacy [48].

## Methods

We conducted a pragmatic [49], single arm, prospective, sequential mixed methods study consisting of a pre-post quantitative design followed by a qualitative evaluation to assess the feasibility [48] of the ARC Model. The study protocol is described in detail in a separate publication [50]. The results from the quantitative aspect of the study are reported here. In 2016, we developed ARC in partnership with a multi-stakeholder team comprised of health planners, patient partners, PCPs, and representatives from community resource centres (the Partnership). The ARC approach was informed by existing evidence and aimed to maximize population reach and facilitate access to a broad range of health and social resources. The study was conducted in Central Ottawa (Canada), a region of approximately 416,202 individuals that includes Francophones (15%), immigrants (27%), visible minorities (27%), and individuals living in low income housing (18%) [51].

### ARC model

The ARC model consisted of social prescribing in which navigation services were integrated. Social prescribing is the process through which primary care providers identify unmet health and social needs in their patients, engage them to act on these, and refer those requiring navigation support to navigation services where they receive support to access the resources they need [52]. The ARC navigation services were delivered to patients referred to the program who agreed to participate in the study. The navigation services are centralized, housed at the research institute, with outreach to patients. Navigation services were offered in person at the practice, at a partner community health centre, or another site designated by the patient, such as a coffee shop or library, or through telephone, email, or texting, according to patient preference. First encounters were encouraged to be in person. The ARC navigator was a non-clinical member of the research team formally trained to provide outreach support to several primary care practices. The ARC navigator used a person-centered approach to understand patient needs, expectations, priorities, and access barriers, establish trust, and provide emotional support to build patient engagement and links the individual to the resource(s) that are best suited for them. At the time when patients exit the program, the ARC navigator returned a brief summary of the navigation work to their referring primary care provider. The ARC model offered an approach for integration between primary care and community.

### Practice recruitment

We introduced the ARC Model to a small group of family physicians at a departmental leadership meeting of the Department of Family Medicine at the University of Ottawa and invited those providing comprehensive primary care services in the Central Ottawa to express interest in participating in the study. Two family physicians, both working under a capitation remuneration model, responded. They were sent a study information package followed by a clinical study information session to which all clinical and non-clinical staff were invited. One physician worked in an interprofessional organization that operated out of two practice sites (IPPs), the other belonged to a non-interprofessional organization operating out of three practice sites (NIPPs). IPPs are designated by the Ontario government to receive funding for allied health professionals such as dieticians and social workers, whereas NIPPs are not [53]. The IPP members were encouraged to use the services of the ARC patient navigator to reduce or offload some of the practice’s own social worker’s navigation responsibilities and assess the value of ARC services.

### Patient eligibility and recruitment

The study was open for recruitment between August 2017 and March 2018. All patients of participating physicians were eligible for the ARC services unless they were in medical distress. Providers were encouraged to use a shared decision-making approach to identify the needs patients wanted to address, then request patients’ permission to be contacted by the ARC team prior to faxing their referral form to the research team. Upon receiving the form, the ARC research assistant contacted the patient, explained the study in detail, requested study consent, and scheduled the first patient visit with the navigator, encouraging an in-person encounter.

### Intervention

Participating practices designated a Clinical-Lead and an Implementation-Lead to oversee the study. The implementation of ARC in primary care practices and approach to ARC navigation services are described in detail in a separate publication [50]. Briefly, practices were given a 30-minute orientation session during which the study processes were reviewed and the breadth of health and social resources available to their patients were highlighted to encourage referrals. They were then asked to implement four intervention elements in a way that best aligned with their existing practice processes to minimize disruption. These consisted of 1) posting study promotional material in the waiting room; 2) completing a referral form containing pre-defined needs categories for individuals agreeing to engage in addressing their need(s), faxing a copy to the study team, and printing another for the patient to formalize the recommendation and promote adherence [10]; 3) making a practice encounter room available where the ARC navigator could meet patients on site at least two half days per week; and 4) establishing their preferred method of communication with the navigator (e.g., charting, faxes). Practices selected their preferred approach to implementing the four elements and were allowed to adjust these during the study if required.

The ARC navigator received a 12-week online and face to face training our team developed specifically for this purpose [54] which included patient-centric communication approach [55,56,57], and motivational interviewing to create engagement and promote patient self-efficacy [58,59,60,61,62]. During that first encounter, the navigator aimed to understand the patient’s needs, expectations, and priorities, identify anticipated access barriers, and develop an engagement plan. Using various strategies, including the use of a regional community and social services helpline (Ottawa211.ca), the navigator identified resource options that best met the patient’s needs and preferences. The navigation services consisted of informational support (e.g., identifying potential resources and explaining the services they provide), instrumental support (e.g., communicating with resource staff to ensure eligibility and alignment with patient needs, completing enrolment/application forms, scheduling appointments, and harnessing additional resources to overcome barriers related to transportation, language, caregiver responsibilities, and other factors), and emotional support (e.g., accompanying the patient to a program’s initial visit; advocating for action on behalf of the patient, offering encouragement, and promoting empowerment) [63,64,65]. The navigator also provided education about existing online and telephone navigation services to support patient empowerment in their self-care. Finally, the navigator ensured adequate exchange of information across the primary care and community sector to promote information continuity and system integration. They provided progress notes to the patient’s PCPs at the start and end of the navigation services and communicated urgent matters if these arose. The navigation services were intended to be episodic and were discontinued when the patient had accessed the needed service(s) or no longer wished to receive navigation support to access these services. This was expected to take no more than 3 months, but support was continued beyond that time frame if required.

### Sample size

We aimed to recruit 4–6 primary care practices and enrol 80 patients to allow us to estimate referral rate, participation level, and success in achieving access with sufficient precision, and assess the seven areas of feasibility [50,66,67].

### Data collection

We conducted rapid cycle evaluations throughout the study to allow for real-time identification of challenges and rapid adaptation to address these issues [68,69]. A first questionnaire was administered after the implementation of the intervention phase and assessed the practices’ experience with the changes and readiness to carry out the intervention. Subsequent questionnaires were administered at 2-months intervals and assessed barriers to engagement in the intervention.

We captured practice context in a baseline survey which the Clinical-Lead completed prior to the study implementation. Participating providers completed a baseline survey which captured their profile, and a post-intervention survey nine months later, just prior to discontinuing the navigation services, which captured their experience with the program.

On the referral form, PCPs recorded the patient’s name, age, sex, contact information, and need(s) to be addressed. The form contained nine pre-established need categories selected by practice based on their anticipated patient needs as well as an “other” category, along with a comment box that allowed for further details to be provided.

Patients completed a telephone survey relating to their socio-demographic and health profile, needs, anticipated barriers to accessing services at the time of enrolment, as well as a baseline measure relating to four dimensions of access [26], and the Patient Activation Measure (PAM) [70] [see appendix 1]. They also completed a post-intervention telephone survey 3 months later which repeated these outcome measures, captured their experience with the service, and assessed whether they had accessed a resource for each of the needs identified at the time of the referral. Since many services have a waiting list, on the recommendation of our patient partners, the definition of access included being on a waiting list or having an upcoming appointment.

Finally, the navigator documented their activities and details of their encounters with patients in a comprehensive electronic navigation charting tool we developed to standardize service delivery and for record keeping, including encounters with patients, practice team members, potential resources.

We grouped the seven areas of feasibility evaluation [48] into four for the purpose of reporting as follows: 1) *Demand*: PCP participation and referral rate, and patient participation rate and profile (needs, barriers, use of navigation services); 2) *Implementation*: adoption of intervention elements, *Adaptation*: changes in the planned intervention approach, *Integration*: incorporation of study related processes into practice flow; 3) *Practicality* and *Acceptability*: navigator time, and provider and patient experience; and 4) *Potential for Efficacy*: Access to resources captured in a post-intervention survey and changes in patient abilities, and changes in PAM from baseline to post-intervention.

### Analysis

We report on all quantitative measures using descriptive statistics. Patient needs were grouped into those related to health behaviour, health, and the SDoH. We assessed whether a participant accessed at least one resource, as well as the number of resources accessed. We analysed changes between pre and post access abilities questions using the Wilcoxon signed rank test for non-normally distributed data, and changes in PAM using the paired t-test for normally distributed data. In post-hoc analyses, we analysed separately the profile of the IPP and NIPP practice participants, demand, and their experience. We analysed the data using SPSS 25.

## Results

The results for the seven areas of feasibility are reported under: I) Demand; 2) Process of Implementation, Integration and Adaptation of ARC model, 3) Practicality and Acceptability of intervention; 4) Potential for efficacy.

### Demand

**Figure 1:**
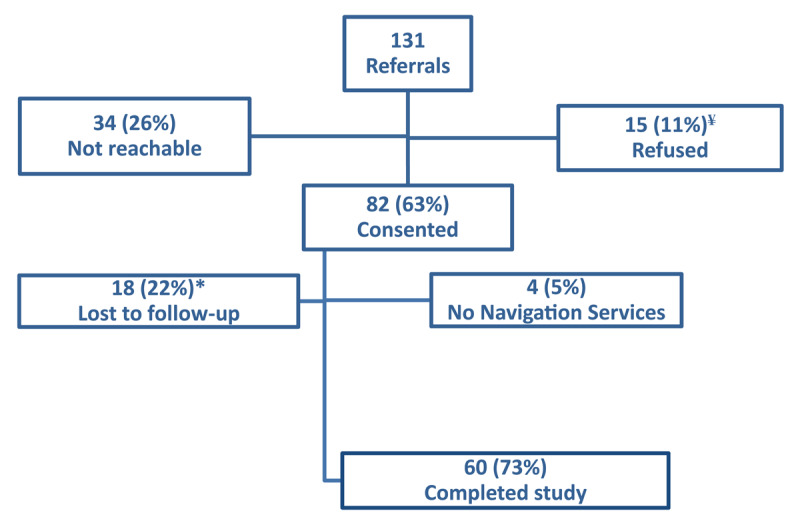
Patient Flow Diagram. * Received navigation services but did not complete the post intervention survey ^¥^ 4 did not have time, 3 did not want the support/had alternative, 2 need no longer present, 4 said would call back and didn’t, 1 didn’t provide a reason, and 1 didn’t want to complete the survey.

One of the two IPPs sites, and all three NIPPs agreed to participate. The IPP was a teaching site involving several residents and other clinicians. All 13 family physicians in the NIPPs and 13 of 16 (81%) staff family physicians (12) and nurse practitioner (1) in the IPP consented (Table 1). Providers in the two models varied in their profile and referral rate. The NIPPs generated most referrals 102 (78%), on average 7.8 per provider. Of the total 131 referrals, 34 (26%) could not be reached, 15 (11%) refused to participate, and 82 (63%) consented (Figure 1). Three patients discontinued the study before completing the baseline survey and another before beginning navigation services. Eighteen (22%) participants did not complete the post intervention survey.

**Table 1:** Primary care provider profile.


ATTRIBUTE	OVERALL	NIPP	IPP

# Consenting (n (%))	26	13 (100)	13 (81%)

# Completing survey (n (%))	21 (81%)	10 (77%)	11 (85%)

Years since graduation (Mean (SD))	28.2 (9.6)	28.1 (9.6)	28.4 (10.2)

Female (n (%))	11 (52)	6 (60)	5 (45)

Canadian Graduate (n (%))	21 (100)	10 (100)	11 (100)

Panel size (mean, (SD))	1,529 (1,166)	1,820 (1,463)	1,239 (749)

Half days worked/week (mean, (SD))	6.1 (2.1)	7.2 (1.7)	5.1 (1.9)

Total number of referrals	131	102	29


**Figure 2:**
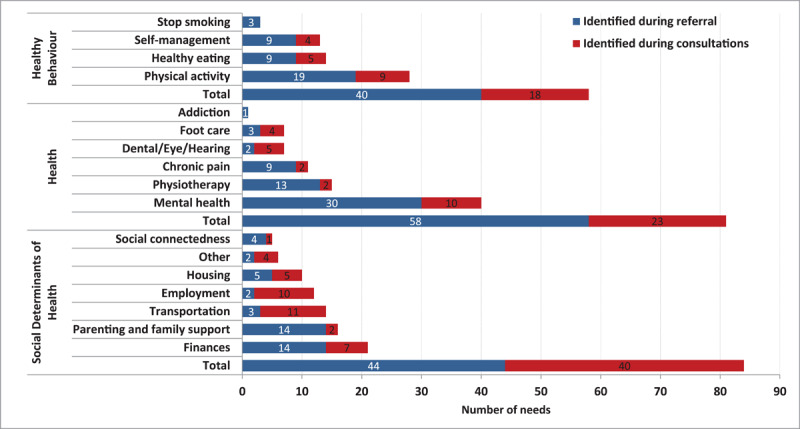
Patient needs as identified by the provider (referral) and during the patient – ARC navigator consultations.

Participant characteristics are shown in Table 2. These demonstrate the presence of social vulnerabilities related to financial needs, low education, and unemployment. On average, 1.8 needs per patient (range 1–7) were recorded on the referral form, and 1.0 (range 0–5) additional need was identified during the navigation services, often (49%) related to the SDoH (Figure 2). The profile of the participants was similar to that of all patients referred with respect to sex (76%/69% participant/all referred) and needs (Healthy Behaviour: 28%/29%, Health (41%/45%), and SDoH (34%/26%).

**Table 2:** Patient Socio-demographic profile (79 participants).


FACTOR	GROUP	NUMBER (%)

Gender	Female	60 (76)

Age (years)	0–49	30 (38)

	50–64	24 (30)

	65+	25 (32)

Language at home	English only	74 (94)

Immigrant	Born in Canada	64 (81)

Financial Situation	Comfortable/Very comfortable	13 (16)

	Modestly comfortable	21 (27)

	Tight/Very tight/Poor	45 (57)

Household income	<$25.000	25 (32)

	$25.000 – $50.000	20 (25)

	$50.000 +	34 (43)

Highest education	University Degree	20 (25)

	Some Post-Secondary	30 (38)

	High school or less	29 (37)

Occupation	Employed	24 (30)

	Unemployed or Unable to work	29 (37)

	Retired/Other	26 (33)

Ethnic background	White (Caucasian/European)	65 (82)

	Black	7 (9)

	Other*	7 (9)

Living alone	Yes	25 (32)

History of anxiety/depression	Yes	54 (69)


* 3 Aboriginal, 2 Latin American, and one each Chinese, Other (unspecified).

**Figure 3:**
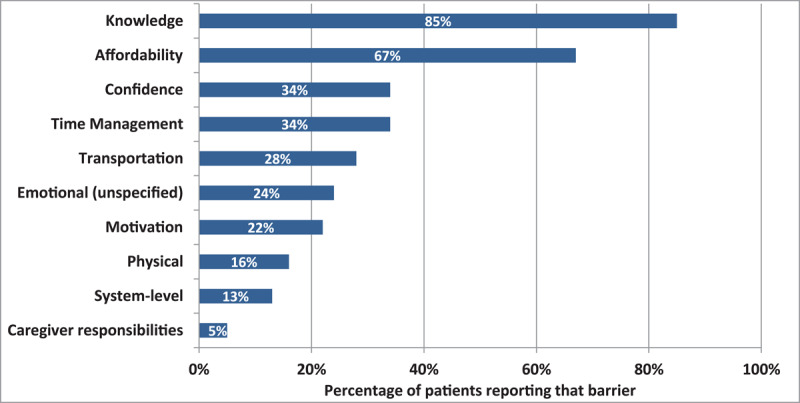
Patient level barriers in accessing community resource(s).

The most cited barriers to accessing services identified at baseline were the lack of awareness of existing resources (85%) and affordability (67%) (Figure 3). Patients declined the help of the navigator for 28 of the 224 needs identified, 22 of which (79%) had been identified by the provider, citing confidence in their ability to access the needed services (57%), and the low priority for the need (29%).

### Process of Implementation, integration, adaptation

The four elements of the ARC Model, listed under “(2.4) Intervention”, were implemented with ease across the four practices with small variations across sites. All practices displayed the promotional material in the waiting room, while the NIPPs requested additional promotional material to post in their examining rooms to remind providers about ARC during their patient encounters. The IPP practice excluded services offered in-house at that site from the referral form, such as dietary counselling. All practices integrated the referral form in their electronic medical records, and opted for fax to communicate with the navigator, with telephone or in person communication as needed. The rapid cycle evaluations identified that providers had an insufficient understanding of the types of services available to their patients and desired more details on the services accessed by their patients. We established a monthly newsletter to showcase various types of health and social resources, and, in consultation with the PCPs, we established a structured navigator template feedback containing their patient’s information from the navigator baseline assessment at the start, and outcome, including services accessed and reason for discontinuation of navigation services. No disruption to practice flow was reported, and no adaptations were required.

### Practicality and Acceptability

**Figure 4:**
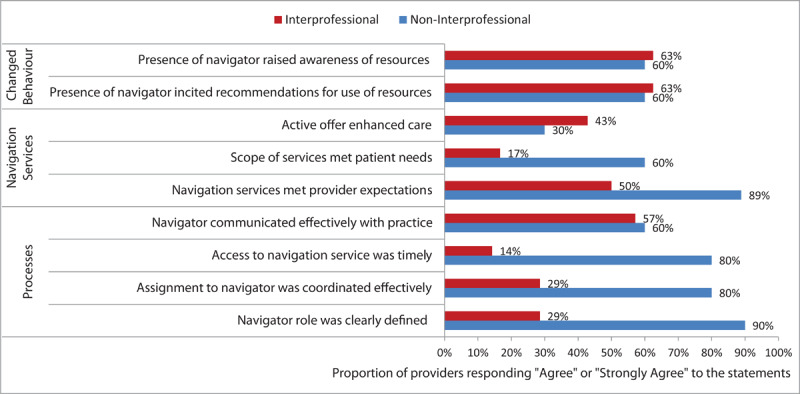
Provider Experience Across Practice Models. Providers were asked to indicate their agreement with statements relating to their experience. Response options ranged from strongly disagree to strongly agree.

On average, the navigator spent 90 minutes on synchronous and 11 minutes on asynchronous communication with patients, and this over an average of 8-week. Distributed across all patients, they spent 22 and 12 minutes communicating with community resources and practice personnel, respectively. The time researching resources was not documented. Figure 4 shows the contrasting experience of the providers in the IPP and NIPPs related to the processes associated with the intervention and to the navigation services themselves. Most providers (60%-63%) in both models reported that the program increased their awareness of existing health and social resources and encouraged them to make referrals.

**Figure 5:**
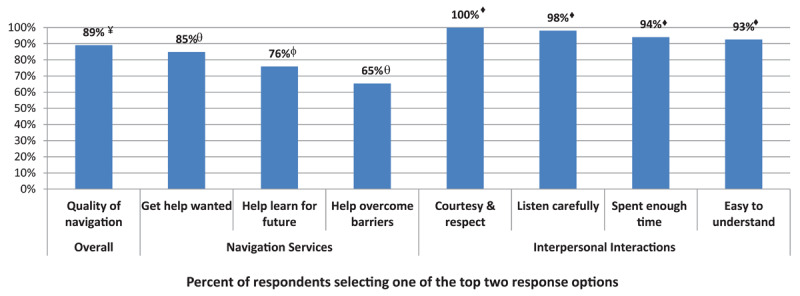
Patient Experience with ARC services. Participants were asked to select their response to statements relating to their experience. Response options were: Good/Excellent vs Poor/Fair^¥^; Usually/Always vs Never/Sometimes^♦^; Yes, generally/Yes, definitely vs No, definitely not/No, not really^θ^; and Yes vs No^φ^.

Patients rated highly the overall quality of the navigation services (89%) and aspects of their interpersonal interaction experience with the navigator (94%-100%) (Figure 5). Most (85%) reported having received the help they wanted and 65% reported that the navigator was able to help them overcome the barriers to reaching the services they wanted. There was no difference in patient experience across practice models (*results not shown*).

### Potential for Efficacy

Thirty-three patients, 40% of the 82 participants (55% of those who completed the study), reported having accessed at least one resource. The likelihood of accessing was relatively similar across participants: females (43%), tight/poor financially, no university degree (47%), living alone (52%), and immigrants (53%). Participants accessed services for 59 (42%) of the overall 142 needs identified. Reasons cited for not accessing a resource were that the need no longer existed (29%), and being too busy (22%), physically unwell (22%), or unmotivated/emotionally not ready (15%). The other reasons (12%) were potentially actionable by the navigator (didn’t like the resource, no resource recommended, affordability). We observed a statistically significant improvement in the individual’s ability to identify the type of professional they needed (p < 0.001), which services were available to them (p < 0.001), and in finding the services they need (p < 0.018), but not in their ability to pay or engage in that service or in the PAM (Table 3).

**Table 3:** Ability scores.


	N	PRE	POST	MEAN DIFF. (SD)	p VALUE

* Ability to seek^♦^ (very easy = 1, Not easy at all = 4)					

In general, how easy is it for you to get health information by yourself when you need it?	52	2.08	2.02	0.058 (0.873)	0.64

How easy is it for you to decide which health professionals you need to see?	51	2.06	1.71	0.353 (0.716)	<0.001

* Ability to Perceive^♦^ (very easy = 1, Not easy at all = 4)					

How easy is it for you to find out which health services you have the right to receive?	46	2.74	2.17	0.565 (0.981)	<0.001

How easy is it for you to find the healthcare you need?	53	2.34	2.02	0.321 (0.956)	0.018

**Ability to pay**^♦^ (Never = 1, Often = 3)					

How often did you not take drugs that were prescribed by a doctor or nurse because of their cost?	53	1.3	1.25	0.057 (0.497)	0.41

How often did you not take laboratory tests or exams that were prescribed by a doctor or nurse because of their cost (like blood draw, X-rays, etc.)?	54	1.06	1.00	0.056 (0.231)	0.083

**Ability to Engage**^♦^ (very easy = 1, Not easy at all = 4)					

How easy is it for you to explain your problems to health professionals?	55	2.05	1.87	0.182 (0.945)	0.16

**Patient Activation Measure** ^¥^	39	2.98	3.07	–0.082 (0.38)	0.18


Mean Diff = Mean Difference. SD = Standard Deviation. For Abilities, positive values represent a reduction in barriers measured at post-intervention measures. For the Patient Activation Measure, negative scores represent a change in the desired direction. * Starred dimensions were hypothesized to be influenced by the ARC navigation services. ^♦^ Analysed using the Wilcoxon Signed Ranked Text. ^¥^ Analysed using Paired t-test.

## Discussion

We demonstrated that the ARC social prescribing and navigation model can readily be integrated in primary care practices, was highly valued by patients and providers, and addresses an important gap in healthcare services in the context where it was studied. It helped link individuals to available health and social resources that address their unmet needs. We evaluated ARC in Ontario (Canada) which offers a universal health care system and in practices where providers are remunerated through capitation. Since the remuneration structure can influence physicians’ behaviour, the model should be evaluated in the fee for service model [71,72].

ARC was readily integrated in primary care practices without disruption to existing workflows. The successful integration of navigation services in primary care can provide clinicians the confidence and capacity to pursue action on their patients’ SDoH [73] and may help reduce the inequities resulting from the differential access to government-funded allied health resources in IPP and NIPP practices [74].

At the time when the ARC Model was developed in the Ottawa region, social prescribing models had not been introduced in Canada. The ARC Partnership selected to introduce an approach whose elements were later found to mirror the social prescribing model used in the United Kingdom [75]. While there is considerable variability in how social prescribing is implemented, it commonly involves the identification of health and social needs in primary care and referral of patients with unmet needs to a navigator referred to as a link worker to support access to the needed resources [52].

Our findings suggest that the practice changes implemented did not contravene to practice usual function, and that the availability of the navigation services encouraged providers in both practice models to engage their patients in addressing their health and social needs. However, the referral rate was considerably lower in the IPP, potentially because their existing allied health professionals were already fulfilling the navigation function. Providers in both models reported that the study enticed them to social prescribe, although the experience with the navigation service was superior in the NIPP. We did not find studies that quantitatively measured the PCPs’ experience with social prescribing.

Patient experience with the navigation program was very positive, and largely responded to their expectations. The reasons provided by patients for not accessing a resource were most commonly related to change in need status, physical wellness or emotional barriers, suggesting that the ARC navigation information and instrumental support helped overcome barriers related to these, but that, at least for some patients, the patient-centred approach and the emotional support was insufficient to overcome lack of patient readiness for action [76,77]. However, the very positive interpersonal relationships with the navigator likely contributed to good compliance amongst those at higher readiness levels. A realist review conducted in social prescribing programs suggests that the navigator contributes to the individual’s social capital which results in better motivation to engage in self-care [78]. Another review highlighted the importance of a good alignment between patient expectation and recommended resources [79]. There are now several navigator training programs [54,80], and future studies should explore what elements of the navigation in the various models are most impactful.

Comparing post intervention to baseline measures suggests that the ARC Model approach improved the individual’s ability to seek care they needed, know what is available to them, and their ability to find these services. Because ARC was applied to address a broad range of needs, we could not evaluate the impact of the Model on health outcomes. Social prescribing and navigation studies have demonstrated the potential for that model to improve quality of life, reduce loneliness and decrease medical services, including emergency room visits in some cases [81,82,83,84,85].

Patient participation rate (63%) was in accord with other navigation studies [86]. Roughly one quarter of patients referred could not be reached, likely reflecting the patient’s lack of readiness for change [76]. While referral was intended to be a joint patient-provider decision, studies indicate that most providers do not assess the individual’s readiness for change before referring them to external resources [87]. A significant proportion of individuals referred to the navigation services (69%) had a history of depression or anxiety. These conditions are associated with a higher risk of undesirable health behaviours, adverse social conditions and poor health [6,88,89,90,91,92,93], as well as greater access barriers related to psychological factors and stigmatization [94,95]. We observed that patients commonly identified additional needs during their navigation encounters, most commonly related to the SDoH; factors that are usually not address or documented in primary care. The use of motivational interviewing by the ARC navigator to help individuals explore their perceived barriers and create motivation for action on their needs [58,59,60,61,62], may have incited additional needs to be identified.

Findings from this feasibility study informed the design of our randomized controlled trial (RCT) of the ARC navigation intervention. Notably, recruitment of non-inter-professional primary care practices was prioritized, and providers were oriented to available community resources using a case study approach that depicts patients’ experience accessing various community resources with the support of navigation services. Future studies should seek to understand how social prescribing and their navigation services can be enhanced. A good inter-personal relationship between the care provider and patient is important to promote compliance with self-care [96].

### Strengths and limitations

Without a control comparison group, preliminary findings, even with statistical significance should be interpreted with caution. The study was conducted in four practices in a single context and the ARC Model needs to be assessed more broadly. However, individuals who agreed to participate in the study had a similar profile to those referred, pointing to a lower risk of participation bias. This study provides a comprehensive assessment of seven areas of the feasibility of an approach and offers relevant insights for the potential future application of the ARC Model.

## Conclusion

The ARC Model is an innovative strategy to support patients’ access to needed resources. The Model is feasible and acceptable to primary care patients and providers and has a demonstrated potential for improving patients’ access to health and social enabling resources. This feasibility study has laid the groundwork for a pragmatic RCT to evaluate the ARC Model’s comparative effectiveness in connecting patients to needed resources, improving health outcomes, and reducing health system costs.
